# Pupil dilation as an index of preferred mutual gaze duration

**DOI:** 10.1098/rsos.160086

**Published:** 2016-07-06

**Authors:** Nicola Binetti, Charlotte Harrison, Antoine Coutrot, Alan Johnston, Isabelle Mareschal

**Affiliations:** 1 Department of Experimental Psychology, University College London, London, UK; 2 CoMPLEX, University College London, London, UK; 3 School of Psychology, University of Nottingham, Nottingham, UK; 4 School of Biological and Chemical Sciences, Psychology, Queen Mary University of London, London, UK

**Keywords:** eye contact, gaze duration, pupillometry, arousal, eye-tracking

## Abstract

Most animals look at each other to signal threat or interest. In humans, this social interaction is usually punctuated with brief periods of mutual eye contact. Deviations from this pattern of gazing behaviour generally make us feel uncomfortable and are a defining characteristic of clinical conditions such as autism or schizophrenia, yet it is unclear what constitutes normal eye contact. Here, we measured, across a wide range of ages, cultures and personality types, the period of direct gaze that feels comfortable and examined whether autonomic factors linked to arousal were indicative of people's preferred amount of eye contact. Surprisingly, we find that preferred period of gaze duration is not dependent on fundamental characteristics such as gender, personality traits or attractiveness. However, we do find that subtle pupillary changes, indicative of physiological arousal, correlate with the amount of eye contact people find comfortable. Specifically, people preferring longer durations of eye contact display faster increases in pupil size when viewing another person than those preferring shorter durations. These results reveal that a person's preferred duration of eye contact is signalled by physiological indices (pupil dilation) beyond volitional control that may play a modulatory role in gaze behaviour.

## 1. Introduction

Eye contact occurs during most animal interactions, often signalling either threat or interest [1,2]. In humans, eye contact provides a non-verbal channel for communicating intentions, regulating interactions and expressing intimacy [3,4]. People show a preference for looking at the eyes compared with other facial attributes [5,6], a feature that is present from a very young age [7,8]. Physiological studies have revealed, using single cell recordings in primates [2,9], and imaging techniques in humans [10,11], the existence of a dedicated neuronal circuitry for the encoding of another's gaze direction that underlies people's accuracy on gaze tasks [1,3,12]. More recently, it has also been shown that under conditions of uncertainty, people tend to perceive another's gaze as being directed towards them; a so-called prior for direct gaze [13]. Taken together, these results highlight the biological significance of gaze processing in human behaviour.

Given the importance of eye contact in human (and non-human) social interactions, as well as the fact that abnormal eye contact is used as a diagnostic tool for clinical symptoms such as autism and schizophrenia [14], it is surprising that ‘normal’ eye contact behaviour remains so ill defined. This is hampered by the fact that gaze behaviour between two people is highly dynamic; therefore, any explicit characterization of gaze behaviour must quantify its spatio-temporal characteristics. For example, the amount of time we are looked at can affect our interpretation of another person's behaviour. Participants receiving longer gazes interpret an observer as having a more favourable opinion of them, and longer gazes are preferred to frequent and short glances [15], yet at the same time overlong gazes [16] or overly short gazes can be discomforting [17–19]. The question therefore is what constitutes a comfortable duration of mutual gaze along this ‘too short’/‘too long’ continuum?

In this study, we examine gaze interactions between a participant and an actor in the following two complementary ways to provide the first quantification of gaze-based interaction durations. First, using behavioural methods, we measure the amount of time an actor can look at a participant without it feeling uncomfortable for the participant (henceforth called ‘preferred gaze duration’, PGD) and examine if this depends on participant personality traits. Second, we relate the PGD to pupil dilation (an index of physiological arousal), motivated by previous reports linking gaze interaction to autonomic responses [20–25]. More specifically, direct opposed to averted gaze stimuli have been observed to elicit increased levels of arousal, as evidenced by skin conductance [26,27] and heart rate measures [28], as well as by increases in blood-oxygen-level dependent (BOLD) signal in the amygdala [29,30]. Similarly, EEG measures of cortical arousal were observed to be modulated both by direct gaze and interpersonal distance [22,24]. Pupil dilation, which represents a reliable index of noradrenergic activity [31–33] and cortical arousal [34], has been directly linked to gaze behaviour by showing increased [3] and prolonged [35] responses to direct gaze stimuli. Here, we explored in greater detail this relationship by linking direct gaze duration preference, assessed on an individual basis, to autonomic activity measured through pupillary response.

Visitors to the London Science Museum judged whether videos of an actor looking at them for different amounts of time felt too long or too short with respect to what they deemed to be comfortable. Behavioural and physiological measures were combined with basic demographics and personality questionnaires to determine whether trait characteristics influenced gazing behaviour.

## 2. Methods and results

### 2.1. Experimental procedure

#### 2.1.1. Participants

We recruited 498 (224 male and 274 female; 56 nationalities) visitors to the London Science Museum, between the ages of 11 and 79 (mean age = 29.9 years; s.d. = 12.3 years; eight participants under 18 years), who volunteered to take part in the study. Written consent was obtained prior to the experiment (given by a guardian for participants under 18 years of age). Participants were informed that they could interrupt the study at any time. The experiment was approved by the UCL Research ethics committee and by the London Science Museum.

#### 2.1.2. Experimental set-up

The study took place at the Live science Pod in the ‘Who am I?’ exhibition in the London Science Museum. The experiment was divided into three sections, for a total duration of approximately 15 min.

*Personality questionnaire.* The Big Five 10-item inventory (BFI-10) [36] was administered on a dedicated set of computers. Each personality trait (extroversion, conscientiousness, neuroticism, openness and agreeableness) was assessed through two items, and item order was randomized across participants.

*Gaze task.* Participants sat at 57 cm from the monitor and head movements were restrained by a chinrest. A protective opaque white screen encased the monitor and part of the participant's head in order to shield the participant from environmental distractions.

**Figure 1.**
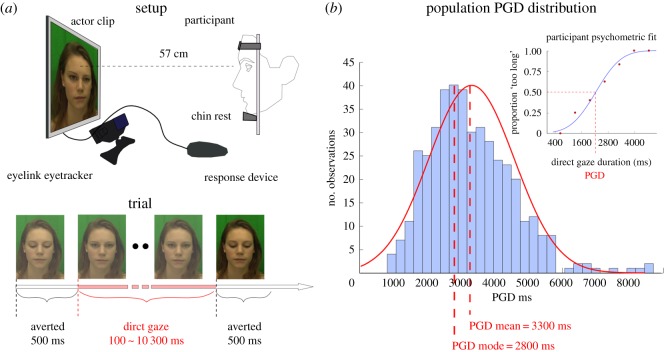
(*a*) Gaze task participant/actor experiment set-up and trial breakdown. Video clips of an actor (randomly selected from a pool of four female and four male actors; one actor per participant) are presented throughout 40 trials. On each clip, the actor directly gazes at the participant for a variable amount of time (between 100 and 10 300 ms, in 300 ms increments, preceded and followed by a 500 ms averted gaze directed at the bottom of the screen). Participants indicate at the end of the clip whether the actor's direct gaze was ‘too short’ or ‘too long’ with respect to what feels comfortable. (*b*) Psychometric fit and preferred gaze duration (PGD) of one participant's proportion of ‘too long’ responses as a function of the actor's direct gaze duration (top right panel) and distribution of PGDs in whole participant population.

*Behavioural task*. Stimuli consisted of video clips of eight different actors (four female, four male; all Caucasian, 20–33 years age range). Video clips were edited so the eye region roughly occupied an equivalent area on the screen and the bridge of the nose (nasion) of all actors was aligned with the screen centre. Actor clips were recorded against a green background in diffuse lighting conditions. Prior to each trial, the nasion position was cued by a black central fixation cross presented on a grey background to ensure homogeneity in participants' first fixation. The stimulus therefore provided a visual reference aiding the binary classification task based on prior experience in real-life dyadic interactions. After the participant's response in each trial, a grey screen with the fixation point appeared for 1 s. In each clip, the actor directly gazed at the participant for a variable amount of time (between 100 and 10 300 ms, in 300 ms increments, resulting in 35 possible clips) preceded and followed by a 500 ms averted gaze directed at the bottom of the screen (figure 1*a*). Clip duration was selected based on randomly perturbed PGD estimates yielded by two interleaved QUEST adaptive staircase routines [37]. On average, participants were shown video clips lasting 3905 ± 1645 ms (which is equivalent to a 2905 ± 1645 ms exposure to direct gaze, having subtracted the 500 + 500 ms of averted gaze in the beginning and end of each trial). Each participant viewed clips of one randomly selected actor (40 clips in total), and indicated with a key press whether the amount of time the actor looked at them felt ‘too short’ or ‘too long’ with respect to what they feel would be comfortable. We provided some context to this by instructing the participant to imagine engaging in a non-verbal interaction with a stranger as can occur on public transport (the tube/metro). Clip duration was selected based on randomly perturbed PGD estimates yielded by two interleaved QUEST adaptive staircase routines [37]. This methodological approach was chosen in order to obtain reliable estimates of PGD with the smallest number of trials possible. A limited number of trials were mandatory in order to minimize fatigue in naive participants and to achieve an optimal testing turnover rate. Through initial piloting, we had preliminarily determined we could get reasonably good psychometric fits of participant responses as a function of direct gaze duration with as few as 40 trials.

*Eyetracking*. Eyetracking data were collected on an EyeLink 1000 (250 Hz; see the electronic supplementary material). The gaze task started once the eye signal could be reliably recorded and eye data calibration was successful.

*Actor rating task.* At the end of the mutual gaze duration task, participants over 18 were asked to rate on a 1–7 scale the attractiveness, threat, dominance and trustworthiness of the actor [38]. Item order was randomized across the participants.

### 2.2. Behavioural results

The randomly perturbed QUEST estimates ensured that each participant was presented stimulus durations which were shorter, or longer, than his/her PGD in roughly equal numbers. The QUEST estimates were binned (bin size varied across participants in order to ensure at 1 tested duration per bin), and we calculated the proportion of ‘too long’/(‘too short’ + ‘too long’) responses per time bin. We fit each participant's responses with a cumulative Gaussian (figure 1*b*, upper right panel). The 50% point of this function yielded an estimate of the participant's PGD and the standard deviation of the underlying Gaussian (s.d.) the participant's sensitivity to differences in direct gaze duration. Only participants with acceptable psychometric fits (lower and upper tails outside of the 0.25 and 0.75 bounds) were further analysed (428 out of 498). We also performed a between subjects one-way ANOVA testing differences in PGD and psychometric curve s.d. on the whole participant population and across participant/actor gender groups (male watching male, MM; male watching female, MF; female watching male, FM; and female watching female, FF). Below, only significant findings are reported (see electronic supplementary material, table S1 for all correlations).

The mean duration of PGD was 3295 ± 706 ms (figure 1*b*), whereas the mean s.d. of the fitted psychometric curves was 703 ms. A one-way ANOVA revealed no significant difference in PGD across participant/actor gender groups (MM, MF, FM and FF; *F*_3,424_ = 1.45, *p* = 0.23, $\eta_{p}^{2}=0.01$), and no significant difference in psychometric curve s.d. across gender groups (*F*_3,424_ = 0.074, *p* = 0.97, $\eta_{p}^{2}=0.001$).

We performed correlations between PGD, s.d., personality scores and face rating scores. These correlations were run on the whole participant population, and run separately for all four participant/actor gender combinations. PGDs significantly correlated with psychometric curve s.d. (*r* = 0.43, *p* < 0.0001), as expected by the scalar property, where variability of time estimates scale proportionally to the duration of a timed interval [39,40]. PGDs significantly correlated with participant age only in male participants looking at female actresses (MF group; *r* = 0.23, *p* = 0.01): PGDs increased linearly with the age of the participant (range: 16–68 years old). For face ratings, only ‘threat’ significantly correlated with PGDs (*r* = −0.13, *p* = 0.005); higher actor threat scores were associated with lower periods of PGD. Surprisingly, no personality trait/PGD correlations were observed, both in the whole participant population and within the four actor/participant gender combinations. Psychometric curve s.d. values negatively correlated with actor attractiveness ratings only in the MM group (*r* = −0.24, *p* = 0.01): higher actor attractiveness scores were associated with smaller psychometric curve s.d. values. Finally, psychometric curve s.d. values correlated with participant personality openness scores in the MF group (*r* = 0.32, *p* = 0.003): male participants with higher openness scores had less steep curves (larger curve s.d. values), perhaps signalling that they were more ‘relaxed’ in their gaze duration classifications.

### 2.3. Eyetracking results

#### 2.3.1. Pupil dilation

We analysed the changes in pupil diameter which are known to reflect autonomic responses [3,35,41] and noradrenergic activity, an important measure of cognitive processing [42,43]. Pupil diameter was expressed on a trial-by-trial basis as a percentage change in diameter with respect to a baseline measure that corresponded to the average pupil size during a 200 ms window preceding the onset of the actor stimulus. Only 200 ms recordings with no loss of signal were accepted as valid baselines. In the instances in which this requirement was not met (14% of trials in whole population), we expressed trial data as a percentage change in diameter with respect to the value recorded in the first sample (33 ms frame) of the trial. Environment luminance was constant throughout the experiment.

**Figure 2.**
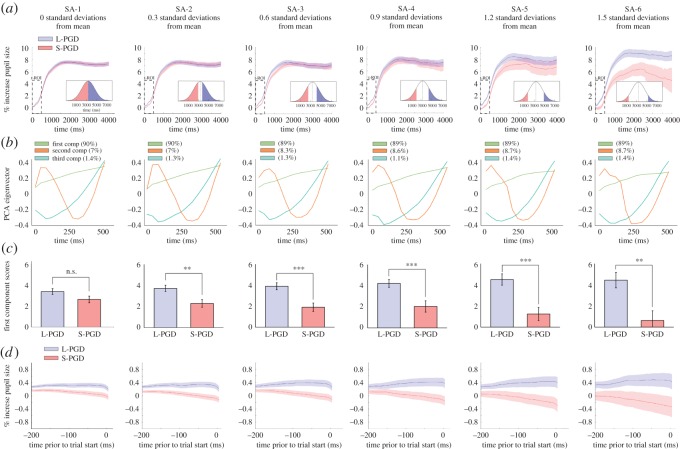
Pupil signal differences between participants favouring direct gaze durations above (longer than) or below (shorter than) the population's mean PGD (L-PGD and S-PGD groups, respectively). (*a*) Participants were sampled at six progressively larger distances from population's mean PGD (six progressively smaller SA—SA 1–6; see figure insets in bottom right corners). Averaged pupil signals for L-PGD and S-PGD groups across each sampling area. Note that error bars (s.e.) progressively increase as the number of participants decreases for greater trial durations. PCA was run on the pupil signal within a 500 ms temporal region of interest (t-ROI; see electronic supplementary material). (*b*) First three components of the PCA run on the first 500 ms of the L-PGD and S-PGD averaged signals. (*c*) PCA mean first component scores between L-PGD and S-PGD groups. Significance thresholds: **p < 0.01; ***p < 0.001. (*d*) Pupil signal during a 200 ms window prior to stimulus onset in L-PGD and S-PGD groups, revealing an anticipatory dissociation in pupil responses between groups.

In order to test differences in pupil dilation as a function of PGD, we assigned the participants to short and long PGD groups (S-PGD and L-PGD, respectively), drawing them from six progressively smaller sampling areas (SAs) of the population PGD distribution (SAs, 1–6, from 0 to 1.5 standard deviations from population mean PGD; figure 2*a* and electronic supplementary material, figure S1*a*). This was done to evaluate whether progressively greater differences in PGD between S-PGD and L-PGD groups (determined by sampling participants at progressively larger distances from the mean PGD), would result in progressively larger differences in pupil signal. We adopted a Functional Data Analysis approach [34,44,45] to test differences in pupil signal between S-PGD and L-PGD groups across time at the six SAs described above. We computed for each participant the average percentage change in pupil diameter across all trials and fit the resulting time series with a b-spline interpolation function [34,44]. At each SA, we assigned participants (based on their PGD) to either the S-PGD or L-PGD group and computed an average b-spline function per group (electronic supplementary material, figure S1*a*). Two-sample *t*-tests were run on the resulting averaged b-spline functions, testing for differences in pupil signal between S-PGD and L-PGD groups across time. At SA-1, we observed no significant difference between S-PGD and L-PGD averaged b-spline functions at any time point. For SA-2 to SA-6, we observe significant differences between S-PGD and L-PGD averaged b-spline functions between 0 and 500 ms (SA-2), 0 and 900 ms (SA-3), 0 and 567 ms (SA-4), 0 and 933 ms (SA-5), 0 and 4400 ms (SA-6; electronic supplementary material, figure S1*b*). Within all these instances, averaged pupil diameter was greater in the L-PGD group than in the S-PGD group. These windows of significant difference seemed to roughly increase in size across SA groupings. In order to further assess differences in L-PGD and S-PGD groups, and relate these differences to other participant variables (age, PGD, face rating scores and personality trait scores), we ran a covariance principal component analysis (PCA) on pupil signal within a fixed 500 ms temporal region of interest (t-ROI), which we defined based on the overlap of windows of significant difference in pupil signal between L-PGD and S-PGD groups across the six SAs as described above (electronic supplementary material, figure S1*c*). The PCA approach reduced the dimensions of pupillary response by identifying a subset of factors along the time axis which account for unique variance in the data [46,47].

We ran a PCA on the % increase in pupil diameter, with participants as observations and the t-ROI time samples as variables. The PCA yielded a series of components, ranked in terms of the percentage of variance in pupil data they explained (the first three components can be seen in the second row of figure 2). We used the elbow criterion [47,48] to determine which components to retain in order to provide a sufficiently accurate summary of the information in the pupil data. For each SA, we ran the PCA on the L-PGD and S-PGD signals within the t-ROI, and found that the first extracted component accounted for most of the variance in the pupil signal throughout all SAs (89 ± 0.64% of variance within t-ROI, figure 2*b*). Thus, we retained only the first component (PC1), which depicts a roughly linear increase in pupil diameter as a function of time, because it explained most of the information. We calculated each participant's first component score (i.e. PC1 score), which represents the coordinate occupied by each participant in PC1 space: the higher the participant's score, the greater the participant's rate of pupil dilation. Finally, we tested difference between L-PGD participants and S-PGD participants to see whether PGD had an influence on rate of pupil dilation. *t*-Tests (unequal variance SA-1 to SA-4, equal variance SA-5, SA-6) run on the first component scores between the L-PGD and S-PGD groups revealed significantly higher first component scores in the L-PGD group for all, but the first SA (SA-1: *t*_392_ = 1.77, *p* = 0.08, *d* = 0.17; SA-2: *t*_297_ = 3.01, *p* = 0.002, *d* = 0.35; SA-3: *t*_206_ = 3.81, *p* = 0.0002, *d* = 0.52; SA-4: *t*_132_ = 3.31, *p* = 0.001, *d* = 0.57; SA-5: *t*_70_ = 3.95, *p* = 0.0002, *d* = 0.94; SA-6: *t*_38_ = 2.89, *p* = 0.006, *d* = 1.08; figure 2*c*). A Kolmogorov goodness-of-fit test showed that L-PGD and S-PGD scores across all SAs were normally distributed. Results were comparable, albeit weaker, for averaged pupil signals obtained when we examined fixations occurring only within the actor's eye regions (see the electronic supplementary material).

**Figure 3.**
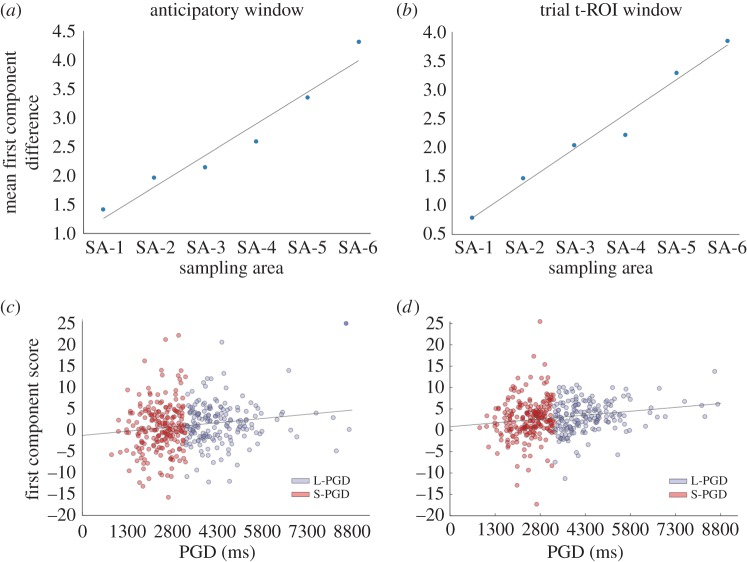
Mean PCA first component score differences between L-PGD and S-PGD groups across the six sampling areas (SA), within a 200 ms anticipatory window preceding the actor face onset (*a*) and within the early 500 ms t-ROI window following the actor face onset (*b*). Mean first component score/PGD correlations related to the pupil signal in the anticipatory (*c*) and early t-ROI windows (*d*).

While the difference between the L-PGD and S-PGD averaged functions notably increased as a function of SA (figure 2*c* and figure 3*b*), the intercepts of these functions appeared to occur at different positions (*y* = 0% increase in pupil diameter), suggesting systematic variations in pupil signals prior to the stimulus onset (figure 2*a*). In order to examine if the differences between the L-PGD and S-PGD functions were due to differences in intercept values, we reran the PCA analysis after vertically repositioning the averaged pupil functions, so their intercepts occurred at *y* = 0. *t*-Tests run on the first component scores between the L-PGD and S-PGD groups revealed weaker but still significantly different first component scores between L-PGD/S-PGD groups for all but the first SA (SA-1: *t*_392_ = 1.04, *p* = 0.29, *d* = 0.11; SA-2: *t*_297_ = 2.06, *p* = 0.04, *d* = 0.25; SA-3: *t*_206_ = 3.2, *p* = 0.001, *d* = 0.46; SA-4: *t*_132_ = 2.48, *p* = 0.01, *d* = 0.43; SA-5: *t*_70_ = 3.02, *p* = 0.003, *d* = 0.72; SA-6: *t*_38_ = 2.66, *p* = 0.01, *d* = 1.02). The L-PGD groups still showed higher first component scores than the S-PGD groups. Therefore, even after eliminating differences in the intercepts, L-PGD and S-PGD pupil signals still differed owing to different rates of pupil dilation following the presentation of the stimulus. To gain further insights into the cause of these differences in intercept values, we applied the PCA approach to percentage changes in pupil diameter within the 200 ms period preceding the onset of the actor face (termed ‘anticipatory window’: figure 2*d*). In this case, pupil diameter was expressed as a percentage increase from an average value recorded between 400 and 200 ms prior to the stimulus onset. Consistent with the results in the t-ROI window following the stimulus onset, we found significantly higher pupil first component scores in the L-PGD group for all SAs in the 200 ms period preceding the stimulus onset (SA-1: *t*_392_ = 2.41, *p* = 0.01, *d* = 0.25; SA-2: *t*_297_ = 2.83, *p* = 0.005, *d* = 0.33; SA-3: *t*_206_ = 2.57, *p* = 0.01, *d* = 0.36; SA-4: *t*_132_ = 2.34, *p* = 0.02, *d* = 0.57; SA-5: *t*_70_ = 2.38, *p* = 0.02, *d* = 0.57; SA-6: *t*_38_ = 1.92, *p* = 0.06, *d* = 0.70), showing an anticipatory dissociation in pupil response between the L-PGD and S-PGD groups (figure 3*a*). We further explored pupil responses in a 600 ms period preceding the stimulus onset (electronic supplementary material, figure S2), and found that the dissociation between L-PGD and S-PGD groups emerges as an anticipatory response to the upcoming trial and is not a result of exposure to the previous stimulus carrying forward.

Pupil signal first component scores (PC1 scores), which summarize for each participant the rate in pupil diameter increase (occurring in the 200 ms anticipatory window preceding the stimulus onset, and in the 500 ms t-ROI window following stimulus onset), were correlated with participant age, PGD, the four actor face rating scores (dominance, threat, attractiveness, trustworthiness), and the five personality trait scores (extraversion, conscientiousness, neuroticism, openness, agreeableness; see electronic supplementary material, table S2). We observed only significant PGD/PC1 score correlations, for both the 200 ms anticipatory window and the 500 ms t-ROI window (anticipatory window: *r* = 0.16, *p* = 0.001; trial *t*-ROI window: *r* = 0.18, *p* = 3.21 × 10^−4^; Bonferroni-corrected critical *p* = 0.0045; figure 3*c,d*). These results showed that PC1 scores increased linearly with preferred period of mutual gaze. Pupil signal PCA first component scores did not correlate with participant age, actor face ratings or participant personality scores (see electronic supplementary material, table S2). We also tested a pupil signal first component score/coefficient of variation correlation (i.e. s.d. scaled by PGD, providing an estimate of error that accounts for scalar variability), which yielded no significant result (*r* = 0.04, *p* = 0.37). We did, however, find a significantly positive correlation between pupil signal and participant psychometric curve variance scaled by PGD (*r* = 0.15, *p* = 0.002), which suggests a nonlinear relationship between response variability and rate of pupil size increase. This positive correlation would imply that participants with a faster increase in pupil diameter have a less strict criterion (greater error) in determining if a period of direct gaze has exceeded or fallen short of a ‘comfortable’ duration.

#### 2.3.2. Fixations: duration, proportion and locations

Given the explicit instruction to evaluate the actor's period of direct gaze, we found that the majority of fixations occurred within the actor's eye region (electronic supplementary material, figure S3*a*). We thus proceeded to study fixation behaviour within three regions of interest (ROIs) defined as (i) left eye, (ii) right eye, and (iii) background (fixations outside eye ROIs). We calculated the duration of fixations (obtained from the data parsing; figure 3*b,c*) and the proportion of fixations (i.e. number of samples in ROI/total number of samples in the trial—accounting for variable trial duration; figure 3*d*) falling within each ROI. We tested differences in fixation behaviour as a function of PGD by correlating PGDs with fixation duration and proportion of fixations across the three ROIs, and found that fixation duration and proportion of fixations are independent of participant PGD.

## 3. Discussion

In this study, we provide the very first large-scale quantification of preferred direct gaze duration and relate this measure to eye tracking, physiological, demographic and personality indices. We find that, on average, participants have a PGD of 3.3 s, consistent with earlier reports obtained in dyadic interactions, i.e. 2.95 [49] and 4.66 s [50]. We also find that changes in pupil size are indicative of a participant's experience of preferred duration of eye contact. Pupil dilation increased at a faster rate in participants who preferred longer periods of direct gaze.

In humans, eye contact serves as a non-verbal channel for communication and social interaction [51–53], and is modulated by a multitude of individual and situational factors [54]. Here, we explored whether preferred duration of direct gaze was modulated by gender, age, face rating and participant personality variables. We did not observe any significant differences in the evaluation of the actor's direct gaze duration across our participant/actor gender combinations, despite gender being suggested to play a role in gaze behaviour [49,55–57]. This might be due to the unidirectional nature of the participant/actor set-up we adopted, which does not fully capture the communicative aspects of a dyadic interaction, or the lack of verbal exchange [58]. We also found no significant variation in PGD across ages within our whole participant population, suggesting that in adults and adolescents (11–17 years), gaze preference is relatively constant. We did, however, find a subtle yet significant interaction between participant age and participant/actor genders: preferred mutual gaze durations increased linearly with age in male participants observing female actresses.

We also explored the impact of threat, attractiveness, dominance and trustworthiness of actor face ratings on PGDs, because these variables can affect engagement or avoidance behaviours [59,60]. We adopted a four-dimensional face classification model that evaluates face features along social dimensions [38], because people tend to spontaneously evaluate personality traits from facial appearance [61]. Direct gaze has been suggested to increase as a function of positive attraction: the number and duration of eye contact instances tend to be larger when observing attractive peers of the opposite sex [62,63]. Gaze also functions to signal threat and dominance during conversations, during defence of personal space and in confrontational scenarios [64,65]. Prolonged gaze in such circumstances increases the likelihood of avoidance behaviours. We find that the only face rating score to affect PGDs in our study was actor threat scores, where higher threat scores were associated with shorter periods of PGD. A possible reason for the lack of influence from the other traits is that they were not scored highly for any of the actors.

There is evidence, albeit some of it conflicting, of a relationship between the amount of mutual gaze and personality traits [66]. A recent study employing a dual eyetracking set-up showed that mutual gaze behaviour correlates with the agreeableness score shared by both parties engaged in mutual gaze [67]. Several studies showed a positive link between gaze and extraversion [19,65,68]; however, others have failed to find this [69–71]. It is possible that capturing any relationship between personality and gaze behaviour is highly dependent on contextual and personal variables that are associated with the experimental set-up [65]. In our dataset, we found no personality/PGD correlation.

Given the explicit instruction to evaluate the actor's period of direct gaze, we found, unsurprisingly, that the majority of fixations occupied the actor's eye regions. We did not detect significant differences in number (proportion) and duration of fixations as a function of PGD within or outside the actor's eye regions. PGD was, however, associated with differences in pupillary response. Emotionally charged events activate parasympathetic pathways which, in turn, engender increases in pupil diameter [72]. Participants that preferred longer periods of direct gaze exhibited greater increases in pupil signal. This dissociation was already evident prior to the stimulus onset, suggesting an anticipatory pupillary response (i.e. trial start was triggered by participant response), and persisted throughout the initial phases of the trial. The degree of pupil dilation evoked by direct eye contact is known to robustly reflect autonomic and noradrenergic activity [3,35,41]. Previous studies have shown that gaze behaviours are typically accompanied by autonomic responses, as assessed through heart rate, galvanic skin response and EEG measures [23,25,65,73]. Specifically, direct gaze has been shown to increase sympathetic activity, both in live dyadic interactions [26,27,74] as well as in participant—static actor image set-ups [28]. Moreover, several studies have documented a positive correlation between direct gaze duration and the amplitude of autonomic responses [21–25]. Here, we further explored this relationship by linking gaze duration preference, assessed on an individual basis, to autonomic activity. We found that the rate of pupil dilation provides a physiological correlate of the subjective preference of direct gaze duration. The PC1 score/PGD correlation implies that one could in theory predict a participant's PGD based solely on the rate of pupil dilation in response to direct gaze stimuli, in the absence of any verbal report.

However, the PGD/pupil dilation correlation might be more generically related to an effect of task difficulty on pupil dilation, which has been frequently documented in the pupillometry literature [75,76]. Stimulus durations were selected with a QUEST staircase: as trials progress and evidence is accumulated through participant responses, the tested durations converge towards the participant's PGD. This implies that participants with longer PGDs were, on average, presented longer direct gaze stimuli than participants with shorter PGDs. Because of the scalar property, where variability of time estimates scale proportionally to the duration of a timed interval [39,40], this suggests that stimuli near longer PGDs are harder to classify than stimuli near shorter PGDs. Therefore, we have the possibility that the faster rate of pupil dilation in the L-PGD group is due to greater task demands relative to the S-PGD group. Two facts, however, work against this possibility. The first is that the difference in pupil dilation between L-PGD and S-PGD groups was observed in the very first 500 ms of the stimulus, whereas effects of task difficulty should be expected to emerge during the decisional phase that follows the encoding of the stimulus [77]. The second is that the L-PGD/S-PGD pupil dissociation anticipates the onset of the stimulus (i.e. prior to the actual start of the timing task). Differences in the stimuli are unlikely to account for the effect, as it would imply that the stimuli, or the testing conditions, systematically differed between the L-PGD and S-PGD groups. The eight actors presented (which might account for differences in the stimuli) were equally distributed across the L-PGD and S-PGD groups. Differences in participant anxiety levels (in response to the stimulus duration) are also an unlikely cause of the effect. Because of the staircase approach, tested durations converged towards each participant's PGD, which implies that all participants were on average presented equally pleasant/unpleasant stimulus durations. Finally, we also controlled for effects induced by time of day by observing no effect on either PGD or PC1 scores.

The modulatory effect of PGD on pupillary responses could depend on different amplitudes in the emotional response elicited by direct gaze between the L-PGD and S-PGD groups. In order to account for the positive correlation between PGD and rate of pupil increase, we could assume that the emotional response evoked by direct gaze is stronger in the L-PGD than in the S-PGD group. This explanation, however, seems at odds with the expectation that direct gaze would probably represent a more discomforting experience for participants with shorter PGDs, and all things being equal, events with negative emotional valences tend to elicit stronger autonomic and behavioural responses [78]. An alternative explanation is offered by recent models detailing the sequential processing of direct eye contact information [53]. Direct eye contact elicits activity in a network of brain areas involved in human social interaction and communication, comprising the fusiform gyrus, anterior and posterior parts of the right superior temporal sulcus, the medial prefrontal and orbitofrontal cortex and the amygdala, i.e. ‘the social brain’ [79–81]. It has been proposed that direct eye contact information is relayed to this cortical network via a ‘fast-track’ subcortical face processing stage, thought to include the superior colliculus, pulvinar and amygdala [53,82–84]. This subcortical stage provides a coarse, fast (150–170 ms latency), context independent processing of direct eye contact information [10]. We could speculate that the dissociation in pupillary response we report as a function of PGD lies in the operation of this ‘fast-track’ stage. This could be reflected in the very early dissociations we observed in pupillary response, compatible with the response latency of this subcortical system, and by the fact that the areas that comprise this fast-track stage are all known to be associated with the noradrenergic system [85–87], of which pupil dilation is a known proxy. This account would suggest that activity within this early eye contact processing stage is enhanced in participants who favour longer periods of direct gaze and who presumably feel more comfortable in engaging in a communicative link. Future studies will be required to specifically uncover how gaze duration preference affects activity in face processing brain circuits.

## Supplementary Material

Methods and Results Supporting Information

## Data accessibility

Raw behavioural and eye tracking data can be found at datadryad.org: doi:10.5061/dryad.dk51c.
