# Supplementary material for: Pupil dilation as an index of preferred mutual gaze duration
Source: R Soc Open Sci. 2016 Jul 6;3(7):160086. doi: 10.1098/rsos.160086 (PMC4968459; doi:10.1098/rsos.160086)
Supplement: Methods and Results Supporting Information [file rsos160086supp1.pdf]

Supporting Information

METHODS

Setup

The experimental setup involved a total 4 computers in order to run 2 participants simultaneously: 2 computers for administering the personality questionnaires, and 2 computers dedicated for the preferred mutual gaze duration task, eye data collection and actor face rating questionnaire.

Upon completion of the questionnaire, participants were invited to occupy one of the two setups. Each setup consisted of a stimulus presentation PC (DELL precision T3500 & DELL precision T3610, both guaranteeing millisecond precision) connected to a 19" LCD monitor (both 1280 x 1024 pixels, 60Hz refresh rate) and an EyeLink 1000 kit (http://www.sr-research.com/).

Task

Stimulus configuration specifics: The point between the eyes (nasion) of all actors was aligned with the center of the screen. Prior to each trial, the nasion position was cued by a black central fixation cross presented on a grey background to ensure homogeneity in participants' first fixation. The stimulus therefore provided a visual reference aiding the binary classification task based on prior experience in real life dyadic interactions. After the participant's response in each trial, the grey screen with the fixation point appeared for 1 second.

Eyetracking: Eye position was calibrated at the beginning of the gaze task with a custom algorithm evaluating fixations on a 3x3 dot array (encompassing 520 vertical x 520 horizontal pixel area). Drift correction was performed every 10 trials on a single central dot. Eyetracking data was collected at 250 Hz (on the EyeLink PCs) and 30 Hz (streamed online to the stimulus presentation machines). The 250 Hz data was used to parse the x / y position signal into fixations with a custom algorithm based on Nyström et al (1). Within trial pupillary dilation was computed from the 30 Hz data, while pre-trial (baseline) was computed from the 250 Hz data. Eyetracking data was successfully recorded in 458 subjects. Position data was evaluated offline on a trial-by-trial basis, and trials with anomalous fixations (fixations consistently outside of the screen area; fixations clearly signalling an incorrect calibration or drift correction) were excluded from the analysis. Both position

and pupil dilation data were further processed through a custom filtering algorithm that substituted signal losses with position / pupil data interpolated from data recorded prior and following the loss of signal. Trials with a loss greater or equal to 18% of total signal were discarded from the analysis. If eye data calibration or recording proved unsuccessful, the experiment skipped directly to the stimulus presentation phase and only behavioural data were obtained.

Since our setup did not involve unrestrained eye fixations, we took several measures to minimize confounds in the pupil signal caused by eye position. One potential confound is represented by the foreshortening of the pupil size (Pupil Foreshortening Effect - PFE) as the eye rotates away from the camera (2, 3). We implemented a PFE correction technique by Hayes & Petrov (4), based on a geometric model that expresses the foreshortening of the pupil area as a function of the cosine of the angle between the eye-to-camera axis and the eye-to-stimulus axis. Using this method, we examined changes in pupil signal across all eye fixation positions (PFE corrected) and changes in pupil signal for fixations exclusively falling with the actor’s eye regions (Left eye or Right eye). We also analysed variations in pupil signal only for fixations occurring within the actor’s eye regions (see *Eye ROI (left & right eye) mean 1st component t-tests, below)*.

RESULTS

Behavioural data

Correlation matrix:

					Actor face rating scores				Participant Big 5 personality traits				
**a)**	Participant Gender	Actor Gender	Participant age	Psychometric curve standard deviation	dominance	threat	attractiveness	trustwothiness	extraversion	conscientiousness	neuroticism	openness	agreeableness
	Both	Both	-0.01(0.88)	0.43(0.00)	-0.07(0.14)	-0.13(0.01)	-0.01(0.84)	0.06(0.21)	-0.06(0.19)	-0.02(0.61)	0.08(0.11)	-0.05(0.29)	-0.07(0.17)
	Male	Male	-0.12(0.22)	0.65(0.00)	0.00(0.96)	-0.10(0.29)	-0.12(0.23)	-0.11(0.24)	-0.04(0.70)	0.03(0.76)	0.10(0.32)	-0.05(0.63)	-0.16(0.10)
	Male	Female	0.27(0.02)	0.19(0.08)	-0.01(0.95)	0.05(0.65)	-0.02(0.83)	0.02(0.89)	-0.01(0.93)	0.09(0.42)	0.12(0.31)	-0.10(0.40)	0.18(0.11)
	Female	Male	-0.06(0.50)	0.35(0.00)	-0.14(0.11)	-0.26(0.00)	0.06(0.49)	0.16(0.07)	-0.06(0.53)	-0.10(0.25)	0.05(0.54)	-0.03(0.78)	-0.16(0.08)
	Female	Female	-0.08(0.41)	0.48(0.00)	-0.10(0.30)	-0.04(0.65)	-0.05(0.63)	0.11(0.24)	-0.15(0.10)	-0.09(0.35)	-0.03(0.77)	-0.04(0.66)	-0.02(0.81)
	Male	Both	0.04(0.57)	0.47(0.00)	-0.00(0.98)	-0.07(0.35)	-0.04(0.60)	-0.04(0.56)	-0.03(0.66)	0.05(0.46)	0.10(0.17)	-0.07(0.35)	-0.03(0.63)
	Male	Both	-0.06(0.32)	0.40(0.00)	-0.12(0.06)	-0.18(0.01)	0.01(0.86)	0.13(0.04)	-0.10(0.13)	-0.10(0.13)	0.02(0.72)	-0.03(0.61)	-0.10(0.11)
	Both	Male	-0.08(0.19)	0.49(0.00)	-0.08(0.22)	-0.20(0.00)	-0.02(0.82)	0.04(0.50)	-0.04(0.50)	-0.04(0.52)	0.08(0.22)	-0.03(0.59)	-0.15(0.02)
	Both	Female	0.12(0.10)	0.34(0.00)	-0.06(0.42)	-0.02(0.82)	-0.02(0.79)	0.08(0.26)	-0.09(0.21)	0.00(0.98)	0.07(0.31)	-0.07(0.30)	0.07(0.32)

					Actor face rating scores				Participant Big 5 personality traits				
**b)**	Participant Gender	Actor Gender	Participant age	PGD	dominance	threat	attractiveness	trustwothiness	extraversion	conscientiousness	neuroticism	openness	agreeableness
	Both	Both	0.00(0.99)	0.43(0.00)	-0.01(0.79)	-0.08(0.08)	-0.01(0.81)	-0.00(0.98)	-0.07(0.13)	0.05(0.26)	0.05(0.27)	-0.05(0.34)	-0.06(0.19)
	Male	Male	-0.07(0.49)	0.65(0.00)	0.06(0.50)	-0.08(0.40)	-0.24(0.01)	-0.18(0.05)	-0.07(0.49)	0.05(0.62)	0.13(0.18)	0.03(0.78)	-0.15(0.13)
	Male	Female	0.06(0.58)	0.19(0.08)	0.11(0.34)	0.05(0.68)	0.10(0.40)	-0.10(0.37)	-0.02(0.87)	0.11(0.34)	-0.05(0.65)	-0.32(0.00)	0.01(0.92)
	Female	Male	0.11(0.21)	0.35(0.00)	-0.13(0.16)	-0.12(0.18)	0.13(0.15)	0.14(0.13)	-0.06(0.50)	-0.06(0.54)	0.07(0.44)	0.05(0.61)	-0.07(0.42)
	Female	Female	-0.18(0.05)	0.48(0.00)	-0.03(0.75)	-0.11(0.26)	-0.08(0.42)	0.08(0.43)	-0.15(0.12)	0.19(0.04)	0.01(0.94)	-0.04(0.64)	0.01(0.94)
	Male	Both	-0.02(0.84)	0.47(0.00)	0.08(0.27)	-0.04(0.57)	-0.08(0.27)	-0.14(0.05)	-0.05(0.51)	0.07(0.34)	0.06(0.44)	-0.12(0.11)	-0.09(0.22)
	Male	Both	0.01(0.86)	0.40(0.00)	-0.09(0.18)	-0.12(0.08)	0.05(0.47)	0.11(0.09)	-0.10(0.14)	0.04(0.53)	0.05(0.48)	0.01(0.89)	-0.04(0.52)
	Both	Male	0.03(0.62)	0.49(0.00)	-0.04(0.56)	-0.10(0.11)	-0.03(0.61)	-0.01(0.94)	-0.06(0.35)	-0.01(0.89)	0.10(0.14)	0.04(0.57)	-0.11(0.10)
	Both	Female	-0.05(0.47)	0.34(0.00)	0.03(0.70)	-0.05(0.52)	0.01(0.91)	-0.00(0.99)	-0.09(0.22)	0.16(0.03)	-0.02(0.83)	-0.17(0.02)	0.01(0.90)

Table 1: Behavioural data correlation matrices. a) Preferred gaze duration (PGD) correlations (r scores, and corresponding p-values in parenthesis) with participant / actor gender combinations,

participant age, psychometric curve standard deviation, actor face ratings and big 5 participant personality trait scores (BFI-10). b) Psychometric curve standard deviation correlations (r scores, and corresponding p-values in parenthesis) with participant / actor gender combinations, participant age, PGD, actor face ratings and big 5 participant personality trait scores.

Eyetracking data

Pupil signal time series T-tests and PCA analysis:

This methodology involves: 1) running t-tests across the L-PGD / S-PGD pupil time series in order to identify differences in the signals within a temporal region of interest (t-ROI: Figure 1), 2) running a PCA with participants as observations and the t-ROI time samples as variables, 3) determining via the elbow criterion (5, 6), the number of components to be retained to obtain a sufficiently accurate summary of the information in the original pupil signal, 4) testing differences in the component scores identified in (3) between the L-PGD and S-PGD groups. This was run only on participants exhibiting an acceptable psychometric fit (see Behavioural Data section) and with a successful pupil signal data recording (N=394).

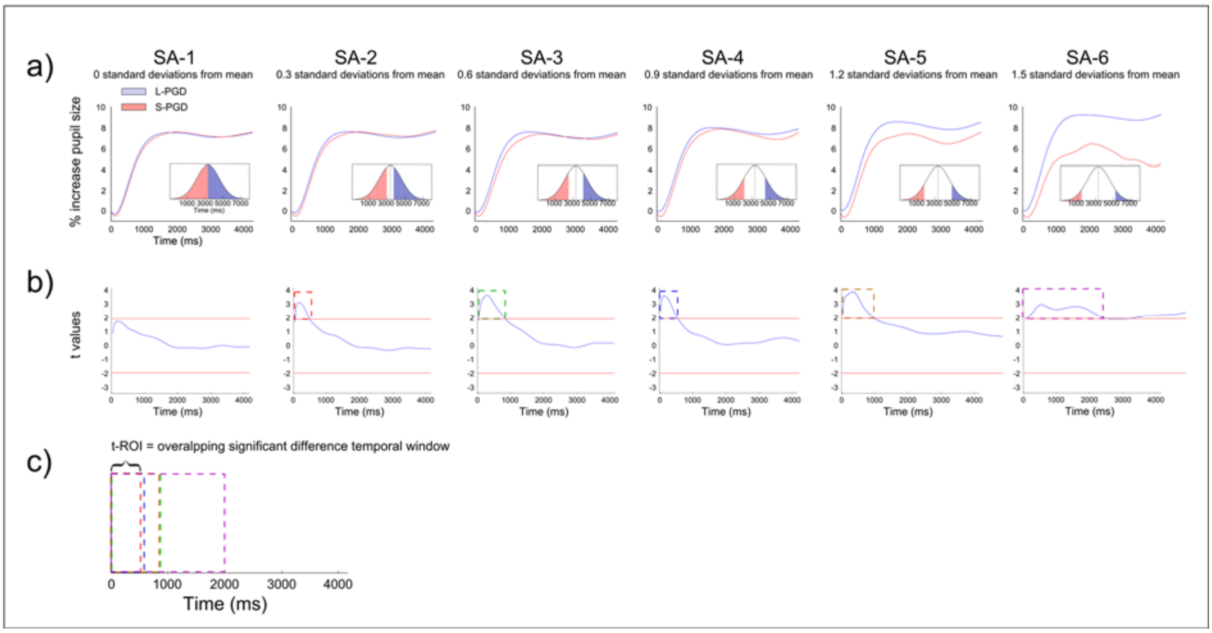

Figure 1: a) Averaged pupil signal b-spline functions for L-PGD and S-PGD groups across 6 sampling areas (SA1 – Si6; i.e. participants were sampled at 6 progressively larger intervals from population's mean PGD). b) L-PGD Vs S-PGD mean pupil signal t-test p-values at each 33 ms time sample. The red lines depict the critical t-value with a p=0.05 threshold: t-values beyond this threshold indicate a significant difference in L-PGD Vs S-PGD mean pupil signal at a given time sample/s. Dotted squares

indicate temporal windows where a significant difference between L-PGD Vs S-PGD mean pupil signal is observed. c) We chose a constant 0 to 500 ms t-ROI in order to directly compare PCA scores across all 6 SAs based on the overlap in pupil signal significant difference temporal windows across 5 out of 6 SAs (2-6).

Eye ROI (left & right eye) pupil signal mean 1st component t-tests:

Difference in pupil signal mean 1st component score between S-PGD and L-PGD groups for fixations recorded in actor Left eye ROI:

Left eye (SA-1: t(392)=1.59, p=.11, d=.17; SA-2: t(297)=2.83, p=.005, d=.34; SA-3: t(206)=3.13, p=.002, d=.45; SA-4: t(132)=2.65, p=.009, d=.47; SA-5: t(70)=1.8, p=.07, d=.44; SA-6: t(38)=1.66, p=.1, d=.64).

Right eye (SA-1: t(392)=1.72, p=.08, d=.27; SA-2: t(297)=1.67, p=.09, d=.29; SA-3: t(206)=2.11, p=.03, d=.45; SA-4: t(132)=1.83, p=.07, d=.49; SA-5: t(70)=1.17, p=.25, d=.43; SA-6: t(38)=1.28, p=.21, d=.73).

Pupil signal in 200 ms time windows prior to stimulus onset:

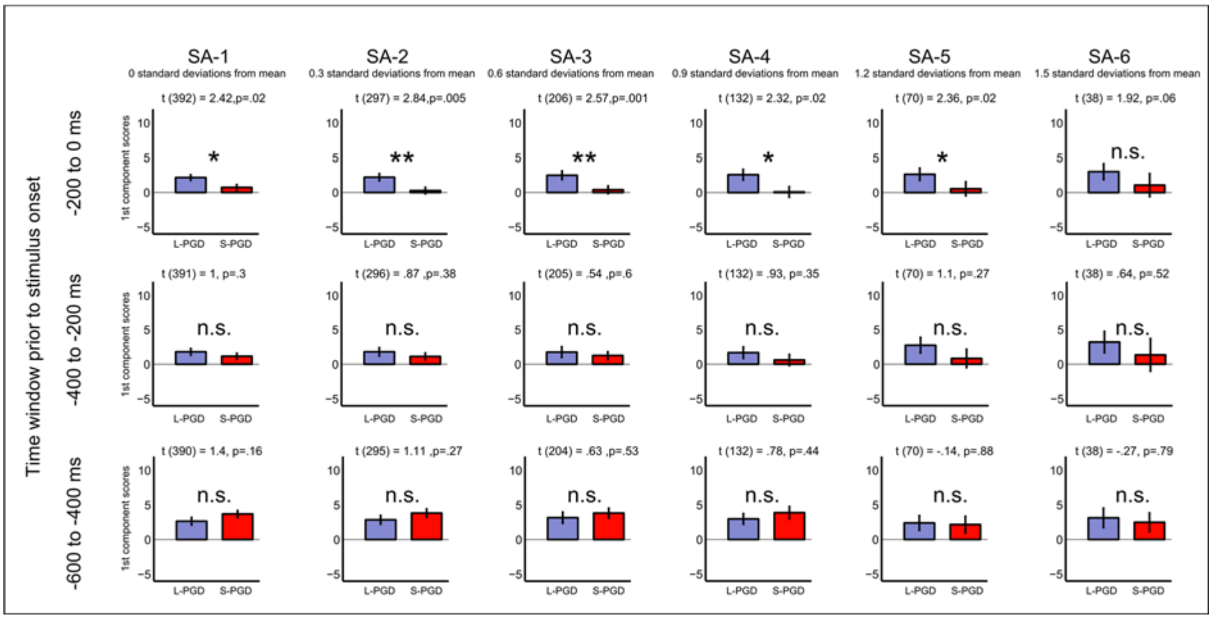

Figure 2: We ran our PCA approach on the averaged pupil signal of the L-PGD and S-PGD groups in three 200 ms windows prior to the stimulus onset (row 1 = -200 to 0 ms, row 2 =-400 to -200 ms

and, row 3 = -600 to -400 ms prior to stimulus onset). Pupil signal in each window was expressed as a % increase in pupil diameter with respect to the mean diameter recorded in a 200 ms period prior to each window (e.g. in the -200 to 0 ms window, pupil size was expressed as a % increase in diameter with respect to the average value recorded between -400 and -200 ms). T-tests were run on the 1st component scores between the L-PGD and S-PGD groups across the 6 SAs. Significant differences in L-PGD / S-PGD pupil signal 1st component scores were only observed in the first 200 ms window immediately preceding the stimulus onset (row 1). The dissociation between L-PGD / S-PGD pupil signals is therefore likely to occur within the 400 ms that precede the onset of the actor face.

Pupil signal 1st Component Score correlation matrix:

			Actor face rating scores				Participant Big 5 Personality scores				
	age	PGD	dominance	threat	attractiveness	trustwothiness	extroversion	conscientiousness	neuroticism	openess	agreeableness
PC1 score	0.09(0.09)	0.18(0.0003)	-0.04(0.47)	-0.03(0.52)	-0.01(0.82)	-0.04(0.41)	-0.07(0.19)	-0.07(0.17)	-0.07(0.19)	0.01(0.84)	-0.01(0.83)

Table 2: Pupil signal PCA 1st component score correlations (r scores, and corresponding p-values in parenthesis; Bonferroni corrected critical p = .0045) with participant age, participant PGD, actor face ratings and big 5 participant personality trait scores (BFI-10).

Eyetracking data: fixation duration, proportions and position:

Fixation durations were entered into a repeated measures ANOVA which revealed a significant effect of ROI (F(2,782)=37, p<.0001, η_p^2=.087). Post-hoc t-test comparisons showed significantly longer fixations in the Right eye with respect to the Left eye ROI (t(393)=-2.53, p=.01, d=.29), and fixations within the Background were significantly shorter than those produced in the Left (t(393)=5.69, p<.0001, d=.29) and Right ROI (t(393)=8.57, p<.0001, d=.43). A repeated measures ANOVA run on the proportion of fixations revealed a significant effect of ROI (F(2,782)=976, p<.00001, η_p^2=.71). Post-hoc comparisons showed that the proportion of fixations was significantly greater in the Left eye with respect to the Right eye ROI (t(393)=4.54, p<.0001, d=.23), consistent with the characteristic asymmetry of fixation patterns reported in the in face processing literature, i.e. “left eye bias” (7). Also, proportion of fixations in the Background ROI were significantly smaller than those observed in the Left eye (t(393)=15.6, p<.0001, d=.78) and Right eye ROI (t(393)=9.91, p<.0001, d=.5).

In order to test differences in fixation behaviour as a function of preferred direct gaze duration, we correlated PGDs with fixation duration and proportion of fixations across the 3 ROIs.

This was run only on participants exhibiting an acceptable psychometric fit (see Behavioural Data section) and with a successful eye data recording (N=393).

Duration fixation did not vary as a function of preferred period of direct gaze (Figure 3E). No significant PGD / fixation duration correlation was observed within left eye (r=-.005, p=.92), Right eye (r=-.007, p=.88) and Background ROI (r=-.01, p=.77).

Similarly, the proportion of fixations did not significantly correlate with preferred period of direct gaze (Figure 3F). No significant correlation was observed within Both eyes (r=-.07, p=.14), Left eye (r=-.06, p=.26); Right eye (r=.006, p=.91) and Background ROI (r=.08, p=.12).

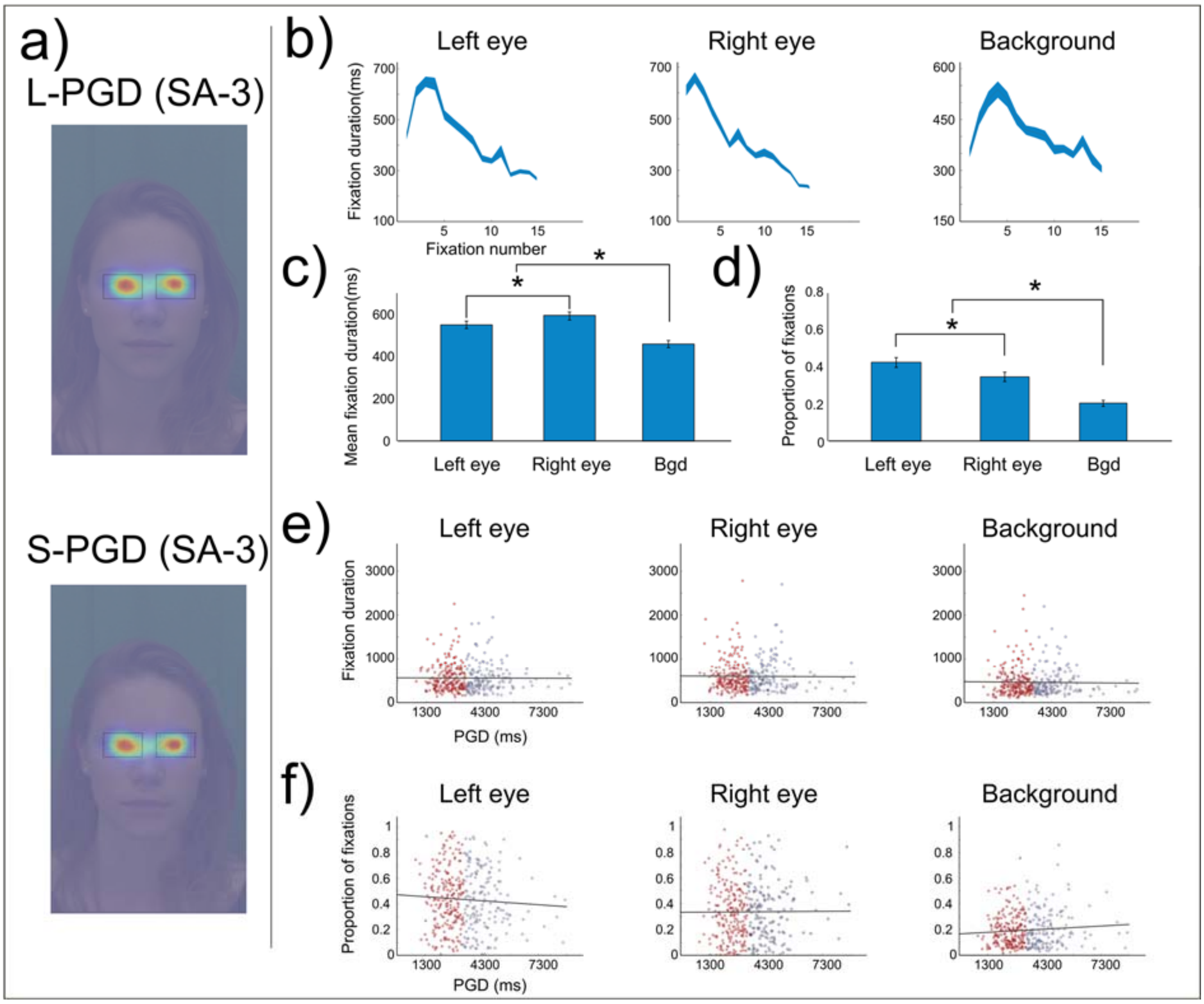

Figure 3: A) Fixation heatmaps of L-PGD and S-PGD groups (sampled at SA-3). Eye regions of interest (ROIs) were defined as 2x3 cm rectangular areas framing each eye. B) Fixation duration (across 1st 15 fixations) within Both eyes, Left eye, Right eye and Background (outside of eyes) ROIs. C) Mean fixation duration across the 4 ROIs. D) Mean proportion of fixations (number of samples inside ROI / total number of samples in trial) across the 4 ROIs. E) Mean fixation duration / PGD correlations across the 4 ROIs. D) Mean proportion of fixations / PGD correlations across the 4 ROIs. Eye regions of interest were defined as a 2x3 cm rectangle encompassing each eye.

References

1. Nyström M & Holmqvist K (2010) An adaptive algorithm for fixation, saccade, and glissade detection in eyetracking data. *Behavior research methods* 42(1):188-204.
2. Jay B (1962) The effective pupillary area at varying perimetric angles. *Vision Res.* 1(5):418-424.
3. Spring K & Stiles W (1948) Apparent shape and size of the pupil viewed obliquely. *The British journal of ophthalmology* 32(6):347.
4. Hayes TR & Petrov AA (2015) Mapping and correcting the influence of gaze position on pupil size measurements. *Behavior research methods*:1-18.
5. Lemercier A*, et al.* (2014) Pupillometry of taste: Methodological guide–from acquisition to data processing-and toolbox for MATLAB. *The Quantitative Methods for Psychology* 10(2):179-199.
6. Shlens J (2014) A tutorial on principal component analysis. *arXiv preprint arXiv:1404.1100*.
7. Gilbert C & Bakan P (1973) Visual asymmetry in perception of faces. *Neuropsychologia* 11(3):355-362.
